# Biochar-seeded struvite precipitation for simultaneous nutrient recovery and chemical oxygen demand removal in leachate: From laboratory to pilot scale

**DOI:** 10.3389/fchem.2022.990321

**Published:** 2022-08-25

**Authors:** Saier Wang, Kechun Sun, Huiming Xiang, Zhiqiang Zhao, Ying Shi, Lianghu Su, Chaoqun Tan, Longjiang Zhang

**Affiliations:** ^1^ Nanjing Institute of Environmental Science, Ministry of Ecology and Environment of China, Nanjing, China; ^2^ School of Civil Engineering, Southeast University, Nanjing, China

**Keywords:** rural RTS refuse leachate, struvite precipitation, biochar seeding, waste management, nutrient recovery, chemical oxygen demand removal

## Abstract

Refuse transfer station (RTS) leachate treatment call for efficient methods to increase nutrient recovery (NH_4_^+^−N and PO_4_^3−^−P) and chemical oxygen demand (COD) removal. In this study, the effects of various operational factors (seeding dose, pH, initial NH_4_^+^-N concentration, and reaction time) on biochar-seeded struvite precipitation were investigated at laboratory and pilot scales. Mealworm frass biochar (MFB) and corn stover biochar (CSB) were used as seeding materials to compare with traditional seed struvite. The maximum NH_4_^+^−N and PO_4_^3−^−P recover efficiency of the MFB-seeded process reached 85.4 and 97.5%, higher than non-seeded (78.5 and 88.0%) and CSB-seeded (80.5 and 92.0%) processes and close to the struvite-seeded (84.5 and 95.1%) process. The MFB-seeded process also exhibited higher COD removal capacity (46.4%) compared to CSB-seeded (35.9%) and struvite-seeded (31.2%) processes and increased the average particle size of the struvite product from 33.7 to 70.2 μm for better sustained release. XRD, FT-IR, and SEM confirmed the orthorhombic crystal structure with organic matter attached to the struvite product. A pilot-scale test was further carried out in a custom-designed stirred tank reactor (20 L). In the pilot-scale test, the MFB-seeded process still spectacularly recovered 77.9% of NH_4_^+^−N and 96.1% of PO_4_^3−^−P with 42.1% COD removal, which was slightly lower than the laboratory test due to insufficient and uniform agitation. On the whole, MFB-seeded struvite precipitation is considered to be a promising pretreatment method for rural RTS leachate.

## Graphical Abstract

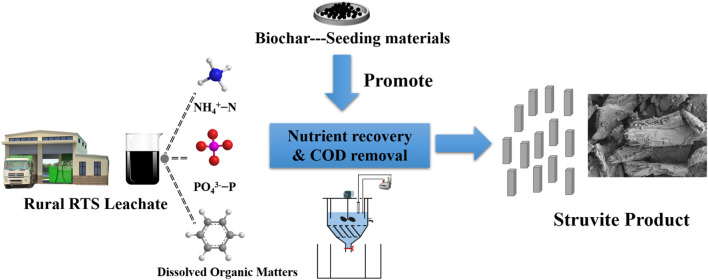

## 1 Introduction

Refuse transfer stations (RTSs), which effectively connect the waste production nodes and the final nodes (landfills or waste incinerators), play a vital role in domestic waste management systems (Chatzouridis and Komilis, 2012; Jia et al., 2021a). In the Chinese rural region, most RTS are small-scale, with a transferring capacity of 50–150 t/d (Ma and Zhao, 2016; Colvero et al., 2020). In RTSs, a liquid waste by-product, refuse leachate, will be inevitably generated during refuse compression and waste collection vehicle washing (Liu and Zheng, 2020). Refuse leachate is complex and hazardous wastewater rich in dissolved organic matter with xenobiotic compounds, ammonia, and heavy metals (Yan et al., 2021; Li et al., 2022). It is estimated that 186 million tons of rural domestic waste are generated annually in China (Patwa et al., 2020). Thus, rural RTSs will ineluctably produce 9.3–18.6 million tons of leachate (5%–10% domestic waste quality) every year. Direct discharge or accidental leachate leakage can cause continuous water and soil pollution, endangering the environment and human health (Gong et al., 2016; Palm et al., 2022). In addition to the high chemical oxygen demand (COD) content (20,000–60,000 mg L^−1^), leachate in the Chinese rural RTS also contains some available nutrients, such as high concentrations of NH_4_^+^−N (200–3,000 mg L^−1^) and small amounts of PO_4_^3−^−P (Lin et al., 2022). Hence, suitable pretreatments for simultaneous nutrient recovery and COD removal of rural RTS leachate are always sought.

Magnesium ammonium phosphate (MAP, MgNH_4_PO_4_∙6H_2_O, commonly known as struvite) precipitation is one of the most common treatments for wastewater (anaerobic digestion, source separation of urine, coking wastewater, etc.) (Ishii and Boyer, 2015; Abel-Denee et al., 2018; Huang et al., 2021; Wu and Vaneeckhaute, 2022). After adding appropriate magnesium sources in a stirred reactor, NH_4_^+^−N and PO_4_^3−^−P can be precipitated as MgNH_4_PO_4_∙6H_2_O, as shown in Eq. 1.

$$Mg^{\mathrm{2+}}\mathrm{+ N}H_{4}^{+}\mathrm{+ P}O_{4}^{\mathrm{3-}}\mathrm{+ 6}H_{2}O\to \mathrm{MgN}H_{4}PO_{4}\cdot 6H_{2}O↓.$$ (1)

The recovered struvite can be used as a slow-release fertilizer in agriculture and forestry, with certain economic benefits (∼$657/ton) (Song et al., 2014). Therefore, struvite precipitation can recycle NH_4_^+^−N, which is not readily biodegradable in leachate, as a reusable resource. In addition, because of its ease of operation, low energy consumption, and operating costs (Gilbert, 2009; Guan et al., 2021b), struvite precipitation could be a suitable treatment for rural RTS leachate. However, complex and changeable water quality characteristics of leachates are the main interference factors of struvite precipitation (Zhao et al., 2013). Many inorganic ions (such as CO_3_^2-^, Ca^2+^, and K^+^) in the leachate will compete for the nucleation of crystals and affect the purity of struvite (Wijekoon et al., 2022). In addition, the crystal size of struvite products has always been another critical bottleneck because larger particles have an enduring effect on the nutrient uptake of plants (Song et al., 2021).

Existing research studies have reported that adding seeding material to precipitation can control the crystallization rate and promote treatment effects (Burns et al., 2016). Seeding material serves as a template through the reaction and its rough surface provides crystal growth sites (Le Corre et al., 2007). Therefore, the specific structure and morphology of seeding material significantly contribute to ion-induced nucleation in the metastable region (Zhang et al., 2021). For struvite precipitation, commercial struvite powder could be the most ideal seeding material, but the cost is high (Yu et al., 2013). Some alternatives, such as zeolite, stainless steel, quartz sand, and borosilicate glass, can also be used as seeding materials. However, the cumulative use of these materials in producing struvite as fertilizer will pose a potential threat to soil health (Perera et al., 2009; Wang et al., 2022).

Among new seeding materials, biochar has low cost and stable properties. With its high porosity and cation exchange capacity, biochar can reduce the thermodynamic hindrance of crystal nucleation (Zhou and Adhikari, 2019). Studies by Zin et al. and Muhmood et al. indicated that biochar seeding of struvite precipitation could improve the efficiency of nutrient recovery and the particle size (Muhmood et al., 2019; Thant and Kim, 2021). Simultaneously, biochar can be applied to the soil to enhance water holding capacity and prevent soil erosion. Combining struvite can compensate for the shortcomings of limited nutrients in biochar, rendering it a high-quality fertilizer (Muys et al., 2021). To the best of our knowledge, few published studies have focused on using biochar as seeding materials to enhance the efficiency of leachate treatment by struvite precipitation.

This study aimed to evaluate the potential of biochar-seeded struvite precipitation as a pretreatment in rural RTS leachate for nutrient recovery and COD removal. Two biochars (mealworm frass biochar and corn stalk biochar) were used as seeding materials and compared with traditional seed, struvite. The effect of various combinations on nutrient recovery efficiency during struvite precipitation, such as seeding dose, pH conditions, initial NH_4_^+^−N concentration, and reaction time was investigated. The particle size, micromorphology, and heavy metal content of the struvite product were also characterized. The fluorescent dissolved organic matter (FDOM) in leachate supernatant was identified and tracked during the precipitation process. The pilot-scale struvite precipitation was further carried out in a custom-designed stirred tank reactor (20 L). This study provided a novel method for the biochar-seeded struvite precipitation and offered valuable new insights for rural RTS leachate management.

## 2 Materials and methods

### 2.1 Characteristics of rural RTS leachate

The fresh leachate used in this study was collected in different seasons from a RTS located in a rural region of Huangshan City, Anhui Province, China (Supplementary Figure S1). The rural RTS has a daily transferring capacity of ca. 100 tons. Prior to being used, the fresh leachate was left to settle for 24 h, and then stored in a refrigerator at 4°C. The typical characteristics of the leachate are presented in Table 1. The pH value of the leachate remained acidic and fluctuated around 4.0. The leachate contained a high concentration of COD in the range of 42,470–50,950 mg L^−1^. The NH_4_^+^−N of the leachate samples ranged from 240 to 625 mg L^−1^ and the PO₄^3-^−P of the leachate samples ranged from 115 to 255 mg L^−1^ throughout the year. Except for Zn (5.87 mg L^−1^) and Cr (0.26 mg L^−1^), the content of heavy metals in the leachate was below the limits of the discharge standard of pollutants for the municipal wastewater treatment plant (GB 18918–2002). The detected VOC concentrations are shown in Supplementary Table S1, and no organophosphorus or organochlorine pesticides were detected in the leachate.

**TABLE 1:** Typical characteristics of leachate used in this study.

Parameter	Leachate	Parameter	Leachate
pH	3.83–4.54	Cu (mg L^−1^)	0.31 ± 0.02
COD (mg L^−1^)	42,470–50,950	Zn (mg L^−1^)	5.87 ± 0.31
N−NH_4_^+^ (mg L^−1^)	240–625	Ni (mg L^−1^)	0.15 ± 0.01
TN (mg L^−1^)	400–1,560	Cr (mg L^−1^)	0.26 ± 0.03
P−PO₄^3-^ (mg L^−1^)	115–255	Pb (μg L^−1^)	658.34 ± 8.61
As (μg L^−1^)	24.21 ± 1.43	Hg (μg L^−1^)	0.03 ± 0.01
Cd (μg L^−1^)	17.20 ± 0.67		

### 2.2 Chemicals and materials

Sodium dihydrogen phosphate (NaH_2_PO_4_, ≥99.7%) and magnesium chloride (MgCl_2_∙6H_2_O, ≥ 99.7%) were purchased from the Sinopharm Chemical Reagent and used as received without further purification. Struvite (NH_4_MgPO_4_⋅6H_2_O, ≥ 98%) was purchased from Aladdin Industrial Co., Ltd (Shanghai, China) and was ground to use as seeding material. Corn stalks were obtained from farms in Jiangsu Province and mealworm frass was obtained from the insect-rearing laboratory in Anhui Province. Corn stalks and mealworm frass were rinsed with deionized water and air-dried. The powdered samples were placed into a sealed crucible and heated to 500°C in a muffle furnace (HPM-2G, AS ONE Corp., Japan), which was kept for 2 h followed by cooling at room temperature under N_2_ atmosphere. Then the obtained corn stover and mealworm frass biochars were ground, sieved through a 0.42 mm mesh size, and used as seeding materials, labeled CSB and MFB, respectively.

### 2.3 Laboratory scale test

MgCl_2_·6H_2_O and NaH_2_PO_4_ were added to the leachate to maintain the equimolar ratio of Mg^2⁺^, NH_4_^+^−N, and PO_4_^3−^−P at 1.2: 1: 1 (Nageshwari and Balasubramanian, 2022). The laboratory test (Supplementary Figure S2) was carried out at 25°C using a six-link mechanical agitator (MY3000-6M, Wuhan Meiyu Instrument Co., Wuhan, China) and an automatic titrator (ZDJ-5B, Shanghai Leici Xinjing Instrument Co., Shanghai, China) as a pH control device. The leachate treatment volume of the laboratory test was 650 ml and the reaction process included a rapid-mix period (10 min, 250 rpm) and a slow-mix period (20 min, 100 rpm) according to suitable stirring rates for struvite precipitation (Wilsenach et al., 2007; Uysal et al., 2010). The dynamic titration method was used during the reaction process to control the pH fluctuation within ±0.05 with 1 M HCl and NaOH.

Different seeding doses (0, 0.1, 0.2, 0.3, 0.4, and 0.5 g L^−1^) were chosen to evaluate the effect of seeding doses on the nutrient recovery under same pH (10.0) and initial NH_4_^+^−N concentration (530 mg L^−1^). The pH value was adjusted from 8.5 to 10.5 with increments of 0.5 to study the effect of pH on nutrient recovery under same seeding dose (0.5 g L^−1^) and initial NH_4_^+^−N concentration (530 mg L^−1^). The initial NH_4_^+^−N concentration was adjusted from 110 to 625 mg L^−1^ to study the effect on nutrient recovery under the same seeding dose (0.5 g L^−1^) and optimal pH for non-seeded (10.0), MFB-seeded (9.5), CSB-seeded (10.0), and struvite-seeded (9.5) processes. The experiments were also conducted for a period of 120 min under same pH (10.0), initial NH_4_^+^−N concentration (530 mg L^−1^), seeding dose (0.5 g L^−1^) and optimal pH for non-seeded (10.0), MFB-seeded (9.5), CSB-seeded (10.0), and struvite-seeded (9.5) processes, and the samples were taken after 5, 10, 15, 20, 25, 30, 60, 80, 100, and 120 min in order to test the effect of reaction time on nutrient recovery.

Approximately 5 ml of the supernatant stood for 60 min after the reaction was drawn with a syringe and filtered through a 0.45 μm membrane to determine the residual contents of NH_4_^+^−N, PO_4_^3−^−P, and COD. The precipitate samples collected after 60 min were freeze-dried at −40°C for 6 h using a freeze dryer (Scientz 10N, Ningbo Scientz Biotechnology Co., Ningbo, China) and then used for further characterizations. All the experiments were replicated twice to improve the reliability of the findings.

### 2.4 Pilot-scale test

**FIGURE 1:**
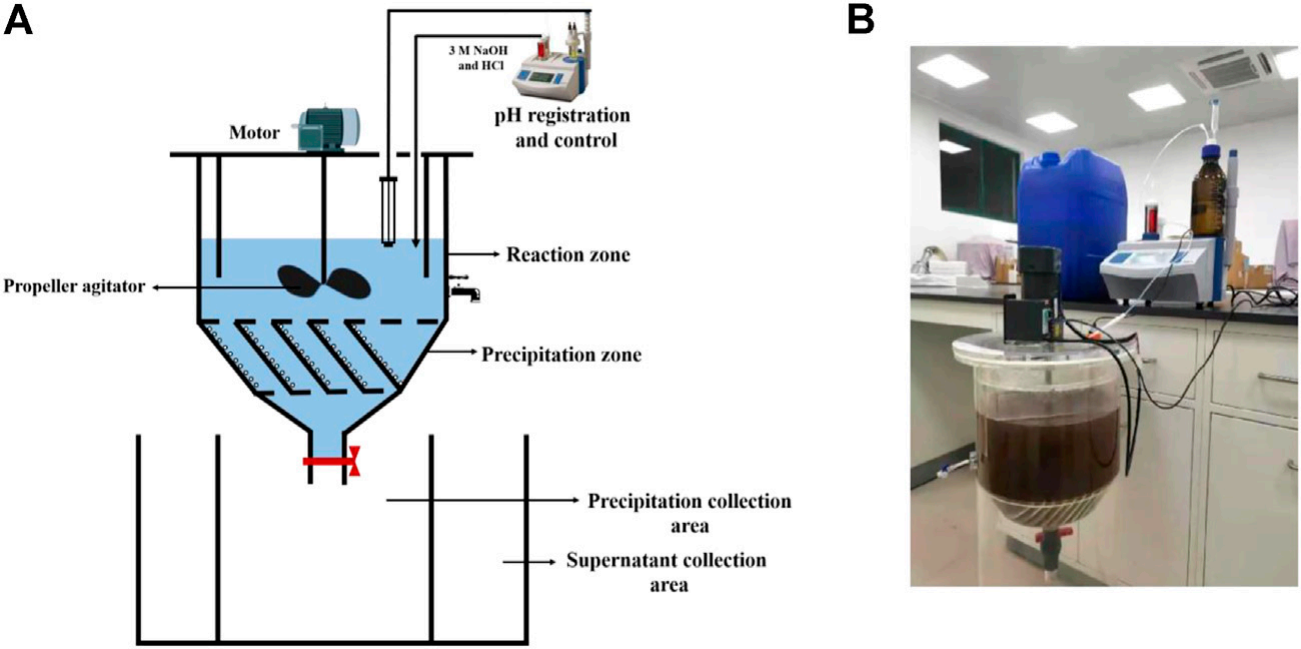
**(A)** Experimental set up of the stirred tank reactor for the pilot test; **(B)** picture of the reactor used in this study.

The pilot-scale struvite precipitation was performed in a custom-designed stirred tank reactor made of plexiglass, as shown in Figure 1. The reactor can be divided into two main units: reaction zone and precipitation zone. The reaction zone had an overall volume of 20 L and was equipped with four baffles to prevent the formation of vortexes and to facilitate mixing. The cone-shaped precipitation zone with an overall volume of 8 L was located below the reaction zone with an angle of 60° between the two zones. This part was equipped with 13 inclined plates (85 mm long, 2 mm thick) to improve precipitation efficiency. The reactor was fitted with a welded metal propeller agitator. Agitation was provided by a motor (4IK25RGN-C, ADDKA, Dongguan, China) and the reaction process included a rapid-mix period (10 min, 250 rpm) and a slow-mix period (20 min, 100 rpm). pH was controlled within ±0.1 with 3 M HCl and NaOH using an automatic titrator (ZDJ-5B, Shanghai Leici Xinjing Instrument Co., Shanghai, China). Precipitation and supernatant collection areas were installed at the bottom part of the reactor and was connected to the reactor through manual valves. The pilot-scale test was carried out under the initial NH_4_^+^−N of 530 mg L^−1^, the seeding material dose of 0.5 g L^−1^, and optimal pH for non-seeded (10.0), MFB-seeded (9.5), CSB-seeded (10.0), and struvite-seeded (9.5) processes.

### 2.5 Analytical methods

The pH values of leachate were measured using a pH meter (S470 SevenExcellence, Mettler Toledo, Switzerland). NH_4_^+^−N, TN, PO_4_^3−^−P, and COD were measured by a multi-parameter water quality tester (GL-900, Shandong Greencarey Instruments Co., China) using the standard methods of the American Public Health Association (Apha and Washington, 2012). Metal content analysis was performed *via* inductively coupled plasma mass spectrometry (ICP-MS, Agilent 7,700, Agilent Technologies Inc., United States) after acid digestion pretreatment.

X-ray diffraction (XRD) was used to analyze the surface-phase composition of the model (D8, Bruker, Germany) using an accelerating voltage of 40 kV and current of 40 mA over a 2θ range of 5°–80°. Graphite monochromatic Cu Kα radiation was used. Fourier-transform infrared spectroscopy (FT-IR) was performed in the 4,000–400 cm^−1^ range (Spectrum 200, Perkin Elmer, United States) to analyze the functional groups within the samples. The morphologies of the samples were characterized *via* scanning electron microscopy (SEM, *Regulus* 8,230, Hitachi, Japan). The adsorption/desorption isotherm of N_2_ at 77 K was obtained by a surface area and pore size analyzer (ASAP 2460, Micrometrics, United States). BET theory was used to calculate the total specific surface area. A laser particle size analyzer (MasterSizer 3,000, Malvern, United Kingdom) was used for particle distribution analysis. For fluorescence excitation–emission matrix (EEM) analysis, the leachate supernatant was filtered through a 0.45-μm membrane, and then each was diluted 100 times with ultrapure water. The fluorescence EEM was measured by an Aqualog fluorescence spectrometer (HORIBA Instruments Inc., Irvine, CA, United States) at an integration time of 1 s, as described in our previous study (Su et al., 2022).

## 3 Results and discussion

### 3.1 Physicochemical properties of seeding materials

**FIGURE 2:**
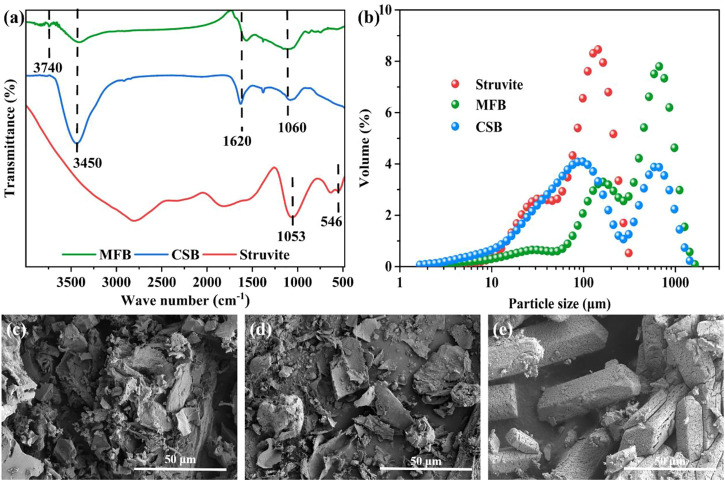
**(A)** FT-IR spectra of MFB, CSB, and struvite; **(B)** particle size distribution of MFB, CSB, and struvite; **(C)** SEM images of MFB; **(D)** SEM images of CSB; and **(E)** SEM images of struvite.

FT-IR was used to obtain the surface functional group information of MFB, CSB, and struvite (Figure 2A). The characteristic peaks at about 3,450, 1,620, and 1,060 cm^−1^ can be observed in the FT-IR diagrams of the two biochars, corresponding to the stretching vibration of −OH, the stretching vibration of C=O and C=C, and the stretching vibration of the ester group, respectively. The peaks at 3,740 cm^−1^ and 700–900 cm^−1^ can be observed in the infrared spectrum of MFB, representing the stretching vibration of the amino group and the out-of-plane bending vibration of the benzene ring (Wu Q. et al., 2022). In the FT-IR diagrams of struvite, broad peaks superimposed by −OH and −NH_4_^+^ (3,500–3,000 cm^−1^), vibrational peaks of phosphate ions (1,053 cm^−1^), and vibrational peaks of Mg−O or Mg−N (546 cm^−1^) can be observed. The N_2_ adsorption test results showed that the specific surface areas of MFB and CSB are 230.8 m^2^ g^−1^ and 42.6 m^2^ g^−1^, respectively (Supplementary Figure S3). A particle size distribution test was also performed on three seeding materials, and the results are shown in Figure 2B. The average particle sizes of MFB, CSB, and struvite are 106, 51.2, and 45.9 μm, respectively. The SEM images showed that the MFB presented a rough, loose, and porous irregular block structure (Figure 2C). The tiny particles on the surface may come from metals (such as K, Ca, and Al) present in the biomass precursors (Chen et al., 2018; Wądrzyk et al., 2021). The smooth flake structure with a length of 5–10 μm and a thickness of 0.5 μm can be seen in SEM images of CSB (Figure 2D). The SEM image of struvite shows the typical morphology of phosphates reported (Figure 2E) (Maqueda et al., 1994).

### 3.2 The nutrient recovery efficiency under various operational factors at the laboratory scale

#### 3.2.1 Effect of the seeding dose

**FIGURE 3:**
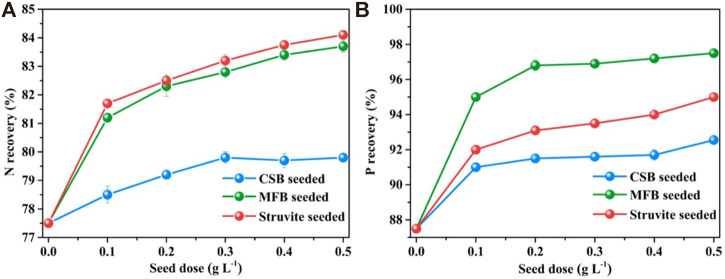
Effect of variation in seeding doses on nutrients recovery through struvite precipitation with various seeding materials from rural RTS refuse leachate **(A)** N recovery and **(B)** P recovery.

The effects of different seeding doses on nutrient recovery efficiency during struvite precipitation process are shown in Figure 3. Without seeding, the NH_4_^+^−N and PO_4_^3−^−P recovery efficiency of leachate treated by struvite precipitation are 77.5 and 87.5%, respectively. When seeding materials were added, the nutrient recovery rate was significantly increased and correlated with the seeding dose positively. The maximum recovery efficiency of NH_4_^+^−N and PO_4_^3−^−P for MFB-seeded (83.7 and 97.5%), CSB-seeded (79.8 and 92.5%), and struvite-seeded (84.1 and 95.0%) processes was obtained at a 0.5 g L^−1^ of seeding dose. Under different seeding doses, the NH_4_^+^−N efficiency of the MFB-seeded process was closed to the struvite-seeded process and was always higher than that of CSB-seeded, while the PO_4_^3−^−P efficiency of the MFB-seeded process was always higher than that of CSB and struvite-seeded processes, probably due to the adsorption of PO_4_^3−^−P by MFB. An increasing seeding dose could provide more active growth sites and lead to secondary nucleation, resulting in a higher nutrient recovery efficiency (Frawley et al., 2012).

#### 3.2.2 Effect of pH

**FIGURE 4:**
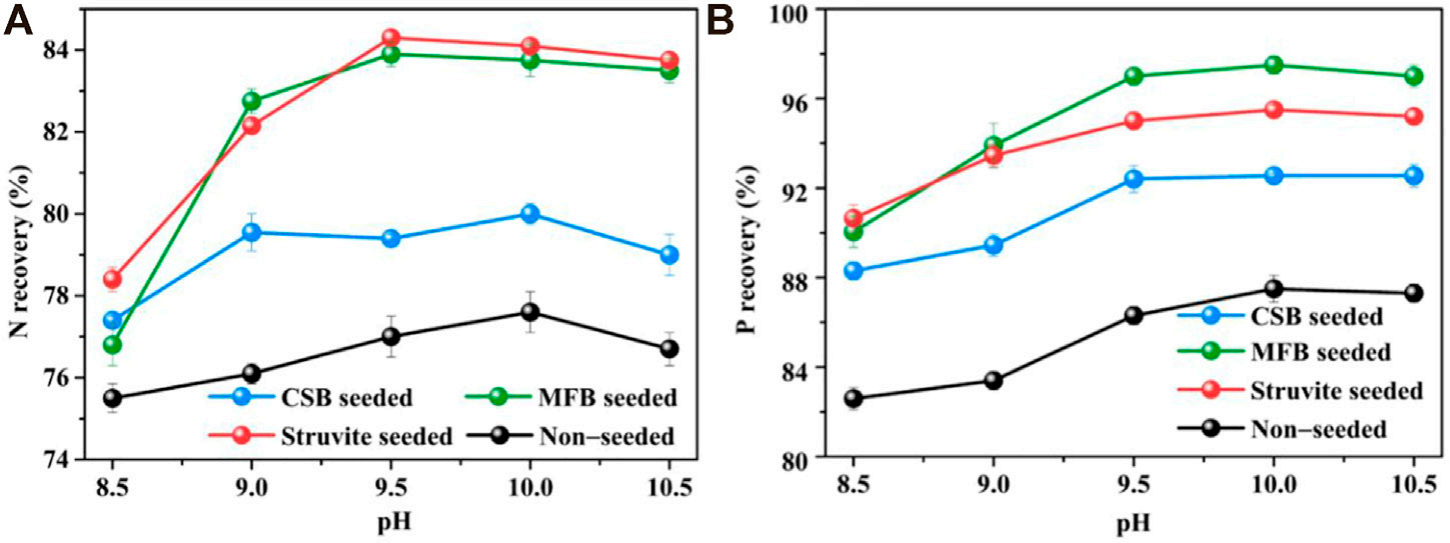
Effect of variation in pH on nutrients recovery through struvite precipitation with various seeding materials from rural RTS refuse leachate **(A)** N recovery and **(B)** P recovery.

As shown in Figure 4, the nutrient recovery efficiency of the non-seeded process increased sharply from 8.5 to 9.5 but fell slightly after 10.0. The maximum NH_4_^+^−N and PO_4_^3−^−P recovery efficiency reached a pH of 10.0. At different pH, adding seeding materials all promoted nutrient recovery of struvite precipitation. For MFB-seeded and struvite-seeded, when the pH was increased from 8.5 to 9.5, the NH_4_^+^−N recovery efficiency significantly increased to the maximum value of 83.9 and 84.3%, with an increase of 7.1 and 5.9%. By comparison, the NH_4_^+^−N recovery efficiency of CSB-seeded reached the maximum value of 80.0% at a pH of 10.0, which was only 2.0% higher than that at a pH of 8.5. The PO_4_^3−^−P recovery efficiency of the three seeding materials all reached the maximum (95.5, 97.5, and 92.6% for struvite-seeded, MFB-seeded, and CSB-seeded, respectively) at a pH of 10.0 and then slightly decreased as the pH continued to rise.

Although the ratio of NH_4_^+^−N and PO_4_^3−^−P in the leachate was adjusted to 1:1 before the reaction, the recovery rate of PO_4_^3−^−P was always higher than that of of NH_4_^+^−N. Existing studies have confirmed that the most suitable pH range for struvite precipitation was 8.0–10.0 with a higher struvite crystallization rate and nutrient recovery efficiency (Roncal-Herrero and Oelkers, 2011; Rodrigues et al., 2022). However, part of the ammonium in the solution is inevitably converted to ammonia in the alkaline range (Huang et al., 2015). Ammonia cannot be precipitated during the struvite crystallization process, so more phosphate reacts with excess magnesium to form by-products such as magnesium phosphate (Mg_3_(PO_4_)_2_·8H_2_O) or potassium metaphosphate (KPO_3_), increasing PO_4_^3−^−P recovery (Huang et al., 2016). Compared with the non-seeded process, the optimal pH of MFB and struvite-seeded processes decreased from 10.0 to 9.5, which has positive implications for cost reduction in practical applications. The possible reason could be that the two seeding materials elevated the driving force during the struvite crystallization process and thus changed the metastable zone defined by pH (Song et al., 2021).

#### 3.2.3 Effect of the initial NH_4_^+^−N concentration

**FIGURE 5:**
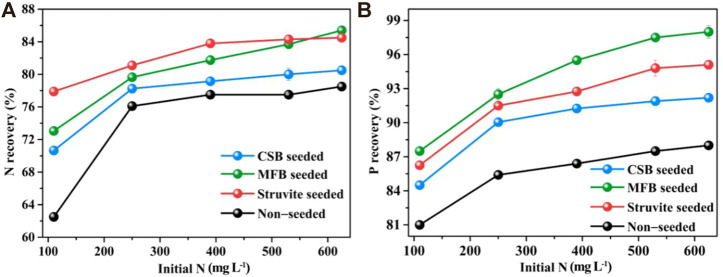
Effect of variation in initial N−NH_4_^+^ concentration on nutrients recovery through struvite precipitation with various seeding materials from rural RTS refuse leachate **(A)** N recovery, **(B)** P recovery.

Since the initial NH_4_^+^−N concentration varies significantly with the seasons, the nutrient recovery efficiency of struvite precipitation under different initial NH_4_^+^−N concentrations was measured. The nutrient recovery of non-seeded, MFB, CSB, and struvite-seeded processes was augmented with increases in initial NH_4_^+^−N concentration (Figure 5). The NH_4_^+^−N recovery efficiency of the MFB-seeded process was slightly lower than that of the struvite-seeded process, and was consistently higher than that of CSB-seeded and non-seeded processes under an initial NH_4_^+^−N concentration of 110–530 mg L^−1^. Due to the dissociation of NH_4_^+^, the NH_3_ content in the leachate will be higher at higher pH. Therefore, the different optimal pH of different struvite precipitation processes could further increase the gap in NH_4_^+^−N recovery efficiency. When initial NH_4_^+^−N concentration was 625 mg L^−1^, the NH_4_^+^−N recovery efficiency of the MFB-seeded process (85.4%) was higher than that of the struvite-seeded process (84.5%). This could be due to the fact that MFB with a higher specific surface area can provide a larger site for crystal growth. Under different initial NH_4_^+^−N concentrations, the PO_4_^3−^−P recovery efficiency all were ranked as MFB-seeded > struvite-seeded > CSB-seeded > non-seeded. Generally, a higher initial NH_4_^+^−N concentration means higher supersaturation of the reaction system. More NH_4_^+^−N can be recovered at higher supersaturation, probably due to the presence and availability of more precipitated constituent ions (Agrawal et al., 2018).

#### 3.2.4 Effect of reaction time

**FIGURE 6:**
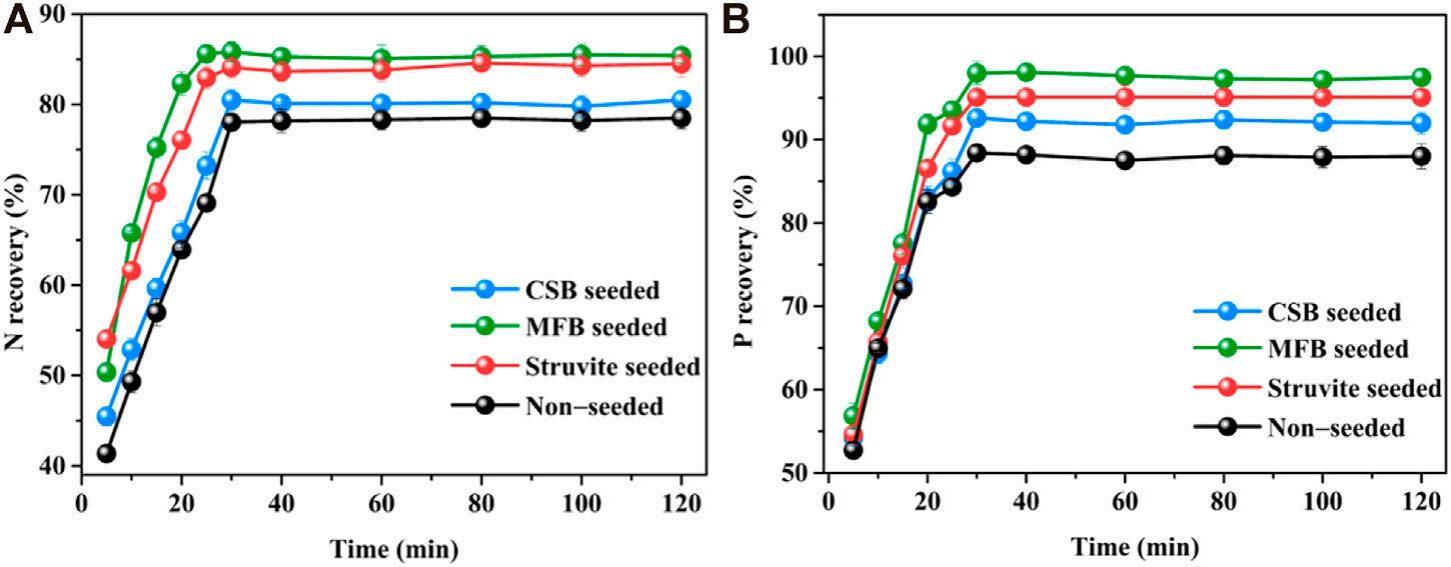
Effect of variation in reaction time on nutrients recovery through struvite precipitation with various seeding materials from rural RTS refuse leachate **(A)** N recovery and **(B)** P recovery.

The changes in nutrient recovery efficiency with reaction time during the struvite precipitation process were also studied. As shown in Figure 6, for the precipitation of non-seeded and seeded struvite precipitation, the reaction proceeded rapidly in the first 5 min. Within the initial 5 min, the recovery efficiency of NH_4_^+^−N and PO_4_^3−^−P for non-seeded, MFB-seeded, CSB-seeded, and struvite-seeded reached 41.3, 50.3, 45.4, 54.0, 56.8, 54.2, and 54.6%, respectively. Because of the gradual decrease in supersaturation, the reaction speed retarded after 5 min until the reaction equilibrium was reached in 30 min and then it leveled off. However, there was also a significant difference in the increase in nutrient recovery from 5 to 30 min during the precipitation of non-seeded and seeded struvite precipitation. For non-seeded, MFB-seeded, CSB-seeded, and struvite-seeded, the recovery efficiency of NH_4_^+^−N and PO_4_^3−^−P increased by 30.5%–37.1% and 35.3%–40.7% from 5 to 30 min, respectively. The nutrient recovery efficiency of the non-seeded and seeded struvite precipitation with the reaction time was also fitted with reaction kinetics (Supplementary Figure S2). For non-seeded and seeded struvite precipitation in leachate, both the NH_4_^+^−N recovery efficiency and the PO_4_^3−^−P recovery efficiency are in line with the quasi-secondary reaction kinetics with an *R* ^2^ > 0.99.

#### 3.2.5 Discussion on nutrient recovery efficiency

Statistically significant differences in nutrient recovery efficiency of non-seeded, MFB-seeded, CSB-seeded, and struvite-seeded processes were analyzed from a two-way ANOVA. ANOVA analysis showed that the nutrient recovery efficiency of the MFB-seeded and struvite-seeded processes was significantly higher than that of non-seeded and CSB-seeded processes (*p* < 0.05). However, nutrient recovery efficiency was not statistically significant between MFB-seeded and struvite-seeded under various operational factors (*p* > 0.05). The linear regression model on the response nutrient recovery efficiency of MFB-seeded process was fitted with respect to the three parameters, namely, seeding dose, pH, and initial NH_4_^+^−N concentration. Supplementary Table S2 depicts the regression model coefficients and associated standard errors (SEs) for the regression coefficients. According to *R* ^2^ values, the correlation between initial NH_4_^+^−N concentration and response variable is stronger than that of pH and seeding dose. The analysis of variance on the regression model is given in Supplementary Table S3. The *p*-value of initial NH_4_^+^−N concentration was less than 0.05 for both NH_4_^+^−N and PO_4_^3−^−P recovery efficiency, indicating that the initial NH_4_^+^−N concentration was significantly correlated with nutrient recovery efficiency. Therefore, for the MFB-seeded process, the initial NH_4_^+^−N concentration seems to be the parameter that most strongly affects the nutrient recovery efficiency.

Compared with the nutrient recovery efficiency with other reported treatments for leachate in Supplementary Table S4, the MFB-seeded struvite precipitation process showed superior performance. Combined with the seeding materials characterization results, the reasons for the enhancement of nutrient recovery by MFB-seeded struvite precipitation could be speculated as follows: 1) higher specific surface area and larger average particle size can provide a larger crystal growth environment (Jin et al., 2021b), 2) irregular and rough surface made it easier for crystals to attach and grow, and 3) the functional groups on the surface of MFB can provide more nucleation sites (Chen et al., 2021).

### 3.3 Characterization of struvite products in laboratory scale struvite precipitation

**FIGURE 7:**
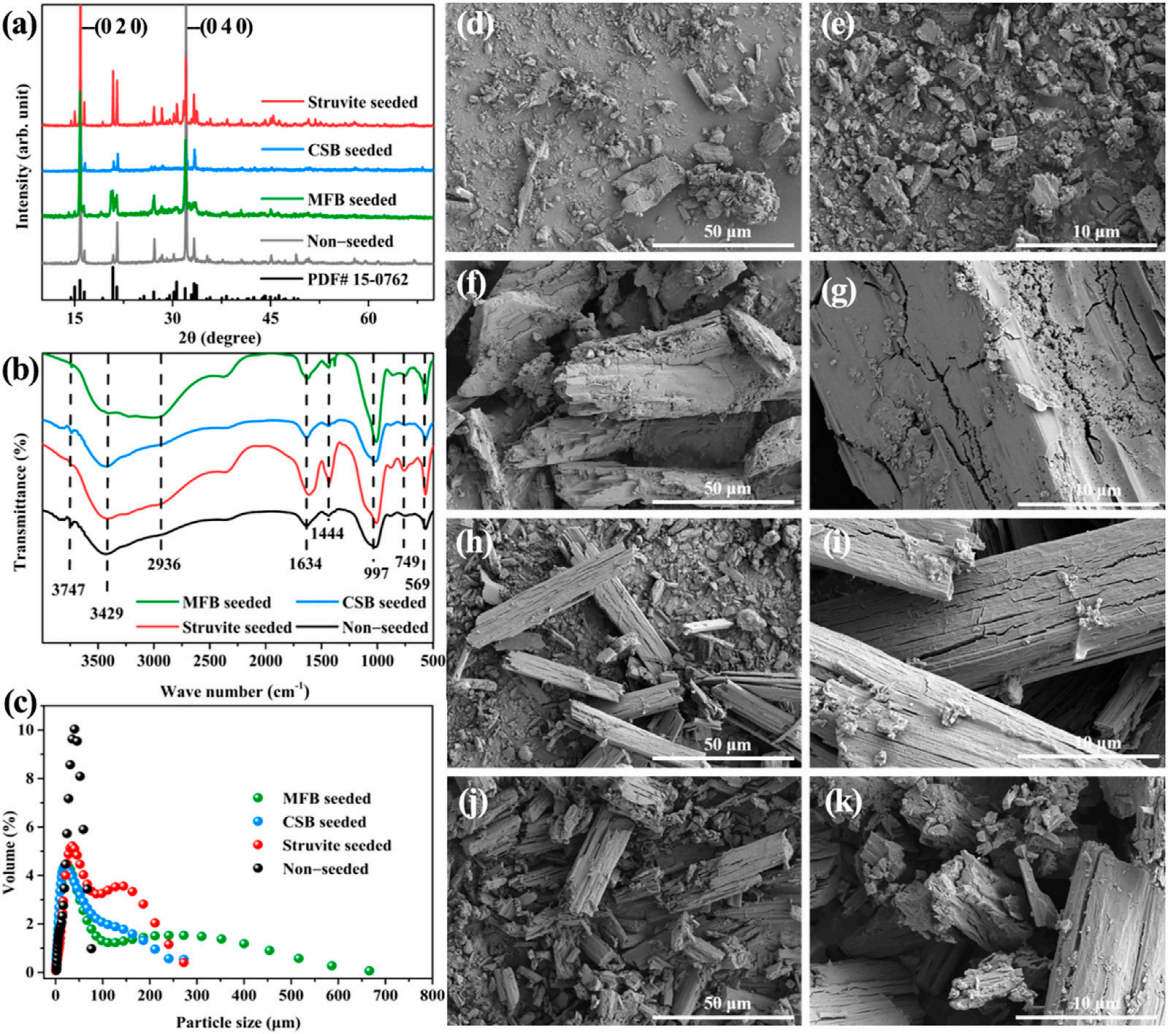
**(A)** XRD patterns of precipitation product; **(B)** FT-IR spectra of precipitation product; **(C)** particle size distribution of the precipitation product; SEM images of the precipitation product **(D)**, **(E)** non-seeded; **(F)**, **(G)** MFB-seeded; **(H)**, **(I)** CSB-seeded **(J), (K);** struvite-seeded.

XRD and FT-IR analyses were carried out to explore the inner structure and surface functional groups of the struvite products obtained after the struvite precipitation of rural RTS leachate. As shown in Figure 7A, the peaks in the XRD spectra of the precipitated products obtained after the non-seeded and seeded struvite precipitation match the standard card of magnesium ammonium phosphate (PDF# 15-0762) quite well, which confirms the struvite crystallizes in the orthorhombic system, space group *Pmn*2_1_ (Zhang et al., 2022). There was a slight difference in the peak intensity between the crystal planes of the struvite product of the non-seeded and seeded struvite precipitation. In particular, the relative strength of (0 2 0) and (0 4 0) crystal planes was relatively high, which may be caused by the adhesion of organic matter in the leachate (Muhmood et al., 2021). The struvite products obtained in each precipitation process showed the same peak positions in the FT-IR diagram (Figure 7B), and were similar to those of other reported struvite products (Suguna et al., 2012). The broad peaks observed near 2,936 cm^−1^ belong to the four N—H stretching vibrations of NH_4_^+^. The peaks at 1,634 cm^−1^ and 1,444 cm^−1^ are attributed to the bending vibration of the N—H bond. The absorption peak at 997 cm^−1^ is attributed to the vibration peak of phosphate ions. By comparison, the moderate-intensity absorption band at 749 cm^−1^ indicates the hydrogen bonding between the coordinated water and the metal–oxygen bond in the precipitated product. The sharp peak at 569 cm^−1^ is attributed to the vibration peak of the bond between Mg and O, N, or halogen (Moragaspitiya et al., 2020).

The particle size of struvite is identified as the most important factor in producing a stable, high-quality, and slow-release fertilizer (Tarragó et al., 2016). Hence, the improvement in the particle size of struvite caused by the seeding material was evaluated (Figure 7C). When non-seeded, the particle size of the precipitated products was concentrated in a range of 1.65–76 μm, and the average particle size was 33.7 μm. After adding 0.5 g L^−1^ of seeding material during the precipitation process, the scope of the particle size distribution of struvite can be significantly broadened. The addition of seeding material also increased the average particle size of struvite. The MFB-seeded process had the most noticeable effect on increasing the particle size, extending the particle size distribution range to 666 μm, and doubling the average particle size. Because the seed can control the crystal growth in the metastable region and effectively inhibit the secondary nucleation (Shaddel et al., 2019), the average particle size also increased to 43.2, 65.5, and 70.2 μm in the CSB-seeded, struvite- seeded, and MFB-seeded processes, respectively.

The morphological properties of the struvite products are shown in Figures 7D–K. Under non-seeded conditions, the struvite precipitation presents a scattered small block structure. The orthorhombic crystal structure can be observed in the SEM image of the struvite product of the seeded struvite precipitation with a size between 20 and 50 μm, which was typical of struvite (Guan et al., 2021a; Han et al., 2021; Wu X. et al., 2022). The struvite product of MFB-seeded treatments has the largest size in the SEM image, which is consistent with the particle size analysis results. The attachment of organic matter can be observed on the surface of struvite products can be observed, which is consistent with the XRD results.

During struvite precipitation, the co-precipitated high concentration of heavy metals will affect the practicality of the struvite product (Liu et al., 2021). There were significant differences in the concentration of heavy metals accumulated in the precipitated products of non-seeded and seeded treatments (Table 2). For example, MFB, CSB, and struvite-seeded had an accumulation effect on both Al and Fe, of which the accumulation effect of CSB was the most obvious. The most considerable accumulation of heavy metals (Zn, Cu, Pb, Cd, Cr, Ni, and Hg) limits for chemical fertilizers clearly defined by the fertilizer art of various countries is listed in Supplementary Table S5. The struvite produced through the seeding had a small accumulation effect on these heavy metals, but it is far below the maximum allowable limit of each country.

**TABLE 2:** Accumulation of heavy metals in the precipitation product.

Parameter	Non-seeded	MFB-seeded	CSB-seeded	Struvite-seeded
Cd (mg kg^−1^)	0.172	<0.01	0.869	1.464
Cr (mg kg ^−1^)	7.033	8.281	13.441	8.652
Cu (mg kg ^−1^)	1.176	3.689	6.106	1.347
Ni (mg kg ^−1^)	3.254	4.480	9.682	4.673
Pb (mg kg ^−1^)	<0.01	<0.01	4.578	1.059
Zn (mg kg ^−1^)	53.825	45.050	38.100	41.475
Hg (μg kg ^−1^)	28.368	89.657	82.490	33.361

### 3.4 COD and FDOM removal in laboratory scale struvite precipitation

**FIGURE 8:**
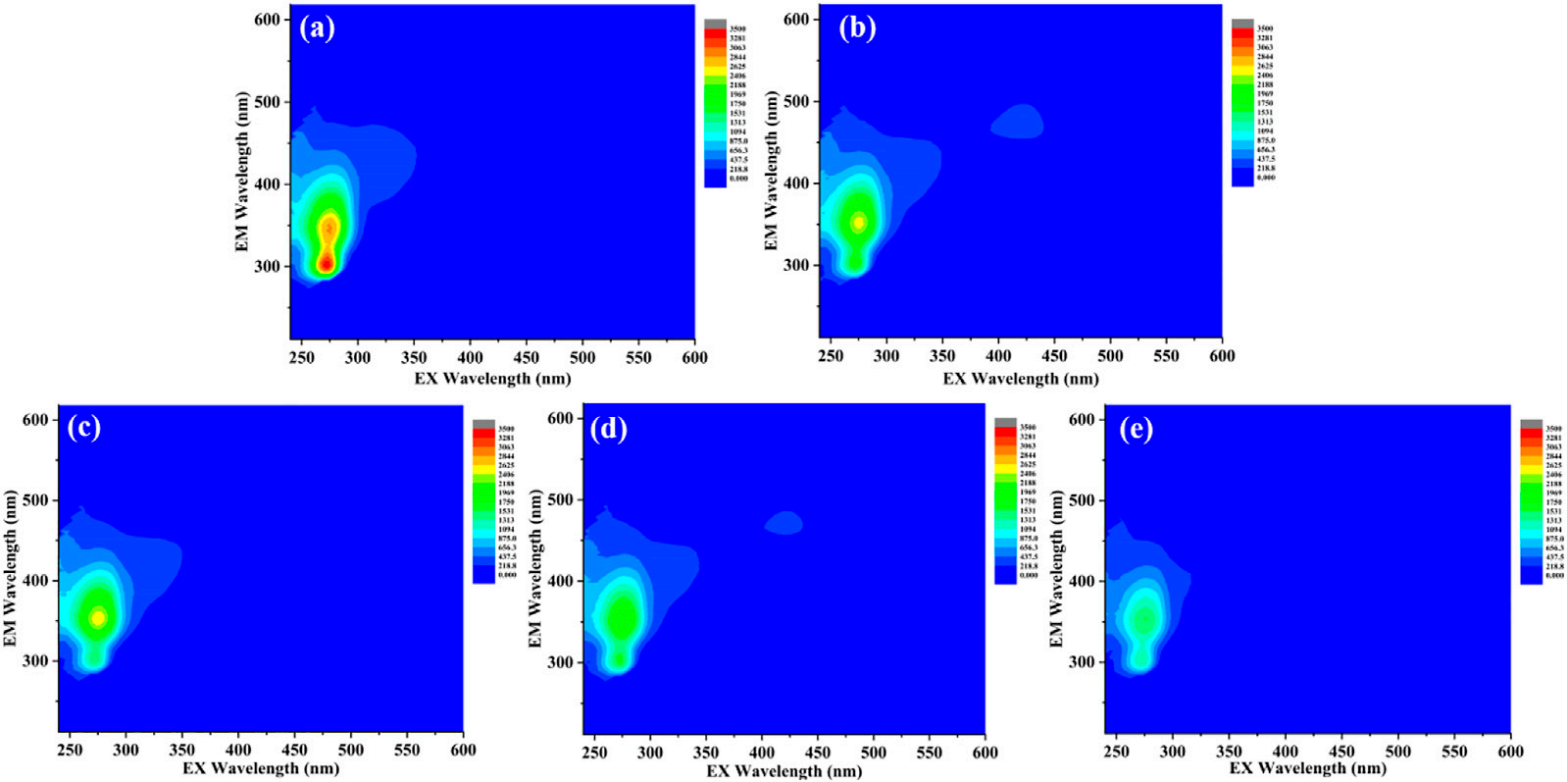
EEM spectra of supernatant **(A)** before the reaction; **(B)** after the non-seeded reaction; **(C)** after the struvite-seeded reaction; **(D)** after the CSB-seeded reaction; and **(E)** after MFB-seeded reaction.

Generally, the large amount of dissolved organic matter (mainly volatile fatty acids and small amounts of hydrocarbons, halogenated hydrocarbons, and ether compounds) contained in the fresh rural RTS leachate resulted in a high COD concentration (Zhang et al., 2015). Hence, COD removal during the struvite precipitation process was investigated. The COD concentration decreased by 25.3% during the non-seeded process. The addition of seeding materials effectively promoted COD removal. After struvite, CSB, and MFB-seeded processes, the COD removal rate reached 31.2, 35.9, and 46.4%. The EEM analysis was carried out to further track the FDOM components in leachate and shown in Figure 8. The main FDOM type in the rural RTS leachate belongs to aromatic protein-like fluorophores (tryptophan and tyrosine), similar to that of fresh landfill leachate (Aftab et al., 2018; Liu et al., 2021). The fluorescence intensities of the peaks observed in the FDOM samples decreased slightly after non-seeded and struvite-seeded processes. However, after the MFB and CSB-seeded processes, the fluorescence intensities decreased significantly, which was consistent with the COD removal. The removal of COD and FDOM could be ascribed to DOM or FDOM co-precipitation with struvite by adsorption onto crystal surface or biochar and partly hydrolysis (Ulu and Kobya, 2020; Ye et al., 2020).

### 3.5 Struvite precipitation in the pilot-scale test

The pilot-scale struvite precipitation using a custom-designed stirred tank reactor (20 L) was performed and the comparison of nutrient recovery and COD removal efficiency to the laboratory scale test is shown in Table 3. Similar to the laboratory test results, struvite-seeded (78.4 and 92.8%) and MFB-seeded (77.9 and 96.1%) processes exhibited higher NH_4_^+^−N and PO_4_^3−^−P recovery than CSB-seeded (73.2 and 89.9%) and non-seeded (69.8 and 85.2%) processes. Compared with the non-seeded process, the NH_4_^+^−N and PO_4_^3−^−P recovery efficiency of struvite, MFB, and CSB treatments increased by 8.6, 8.0, and 3.4, and 7.6, 9.9, and 4.7%, respectively. However, NH_4_^+^−N and PO_4_^3−^−P recovery during each pilot-scale test dropped by 5.8%–7.7% and 1.4%–2.3% compared to laboratory test. The COD value of the leachate before the pilot-scale test was 45,360 mg L^−1^, and its changes were additionally tested during the pilot-scale test. For the non-seeded, and CSB, MFB, and struvite-seeded processes, the COD value after the pilot-scale test was reduced by 22.2, 31.8, 42.1, and 29.2%, respectively. The MFB-seeded process also showed the best COD removal capacity, further verifying that the adsorption of organic matter in this process. Although COD removal could facilitate the subsequent treatment of the leachate, the attachment of organic matter makes the toxicity and fertilizer effect test of the struvite products still warrant more research. The agitation conditions in the pilot-scale test were almost identical to the laboratory test, while the agitation power may be insufficient and uniform to cause the decrease in nutrient recovery and COD removal efficiency when scaling up from the laboratory to the pilot scale.

**TABLE 3:** Performance of the pilot-scale test during non-seeded, MFB-seeded, CSB-seeded, and struvite-seeded processes.

Pilot-scale test	NH_4_^+^−N recovery	PO_4_^3−^−P recovery (%)	COD removal (%)
Non-seeded	69.8%	85.2%	22.2%
MFB-seeded	77.9%	96.1%	42.1%
CSB-seeded	73.2%	89.9%	31.8%
Struvite-seeded	78.4%	92.8%	29.2%

As shown in Supplementary Table S6, an economic analysis of the MFB-seeded precipitation process was carried out. In this assessment, without taking into account the fixed costs of the reactor (∼$450), the costs of chemicals, water and electricity, and profit of struvite product have been considered in the calculations. In particular, the electricity cost included the energy consumption of the muffle furnace for the production of MFB. After calculation, the total cost of the MFB-seeded process for RTS leachate treatment was $5.22/ton. After 20 L pilot test, filtering and drying the precipitate can yield ∼150 g of the struvite product. If the profits of struvite products ($4.73) were deduced from $5.22/ton, $0.49/ton can be obtained as the net cost of the MFB-seeded precipitation process. Overall, the results of the pilot-scale test further validated the MFB process could be a potential pretreatment method for rural RTS leachate.

## 4 Conclusion

In this study, the effects of various operational factors (seeding dose, pH, initial NH_4_^+^-N concentration, and reaction time) on biochar-seeded struvite precipitation as pretreatment for rural RTS leachate were investigated at the laboratory and pilot scale. Two biochars, MFB and CSB, were used as seeding materials, while the traditional seed, struvite, was used for comparison. The maximum NH_4_^+^−N and PO_4_^3−^−P recover efficiency of the MFB-seeded process can reach 85.4 and 97.5%, higher than non-seeded (78.5 and 88.0%) and CSB-seeded (80.5 and 92.0%) processes and close to the struvite-seeded (84.5 and 95.1%) process. The MFB-seeded process also exhibited higher COD removal capacity (46.4%) compared to CSB-seeded (35.9%) and struvite-seeded (31.2%) processes and increased the average particle size of the struvite product from 33.7 to 70.2 μm for a better sustained release. XRD, FT-IR, and SEM confirmed the orthorhombic crystal structure with organic matter attached of the struvite product. A pilot-scale test was further carried out in a custom-designed stirred tank reactor (20 L). In the pilot-scale test, the MFB-seeded process still spectacularly recovered 77.9% of NH_4_^+^−N and 96.1% of PO_4_^3−^−P with 42.1% COD removal, which is slightly lower than the laboratory test due to insufficient and uniform agitation. The toxicity and fertilizer effect test of the struvite product still require further study. Considering the nutrient recovery and COD removal effects and cost, MFB-seeded struvite precipitation could be a promising pretreatment method for rural RTS leachate.

## Data Availability

The original contributions presented in the study are included in the article/Supplementary Material; further inquiries can be directed to the corresponding authors.
