# Supplementary material for: Biochar-seeded struvite precipitation for simultaneous nutrient recovery and chemical oxygen demand removal in leachate: From laboratory to pilot scale
Source: Front Chem. 2022 Aug 25;10:990321. doi: 10.3389/fchem.2022.990321 (PMC9452965; doi:10.3389/fchem.2022.990321)
Supplement: Supplementary file 1 [file DataSheet1.docx]

**Electronic Supplementary Information**

**Biochar seeded struvite precipitation for simultaneous nutrient recovery and COD removal in rural RTS leachate: from laboratory to pilot scale**

Saier Wang, ^a^ Kechun Sun, ^b^ Huiming Xiang, ^a^ Zhiqiang Zhao, ^a^ Ying Shi, ^a^ Lianghu Su, ^a,^ ^*^ Chaoqun Tan, ^b, *^ Longjiang Zhang ^a^

^a^ Nanjing Institute of Environmental Sciences, Ministry of Ecology and Environment, 8 Jiangwangmiao Street, Nanjing 210042, P.R. China

^b^ School of Civil Engineering, Southeast University, Nanjing 210096, P.R. China

* Corresponding author

E-mail: sulianghu@126.com (L. Su), tancq@seu.edu.cn (C. Tan)

**Contents**

[**1. Figures** 3](#_Toc109832327)

[Fig. S1. Scene photos and geographic location of the refuse transfer station 3](#_Toc109832328)

[Fig. S2. Photo of batch experimental device 4](#_Toc109832329)

[Fig. S3. Adsorption-desorption isotherm of (a) MFB, (b) CSB. 5](#_Toc109832330)

[Fig. S4. Pseudo-second-order kinetic fitting of (a) N recovery; (b) P recovery. 6](#_Toc109832331)

[**2. Tables** 7](#_Toc109832332)

[Table S1. Volatile Organic Compounds (VOCs) content of laeachate 7](#_Toc109832333)

[Table S2 Linear regression models for nutrient recovery efficiency of MFB-seeded process 8](#_Toc109832334)

[Table S3 Analysis of variance (ANOVA) for the linear regression model 9](#_Toc109832335)

[Table S4 Comparison of nutrient recovery efficiency with other reported treatment for leachate 10](#_Toc109832336)

[Table S5. Heavy metals limit in fertilizers among various countries 11](#_Toc109832337)

[Table S6 Economic analysis of MFB seeded struvite precipitation process 12](#_Toc109832338)

[Reference 13](#_Toc109832339)

**1. Figures**


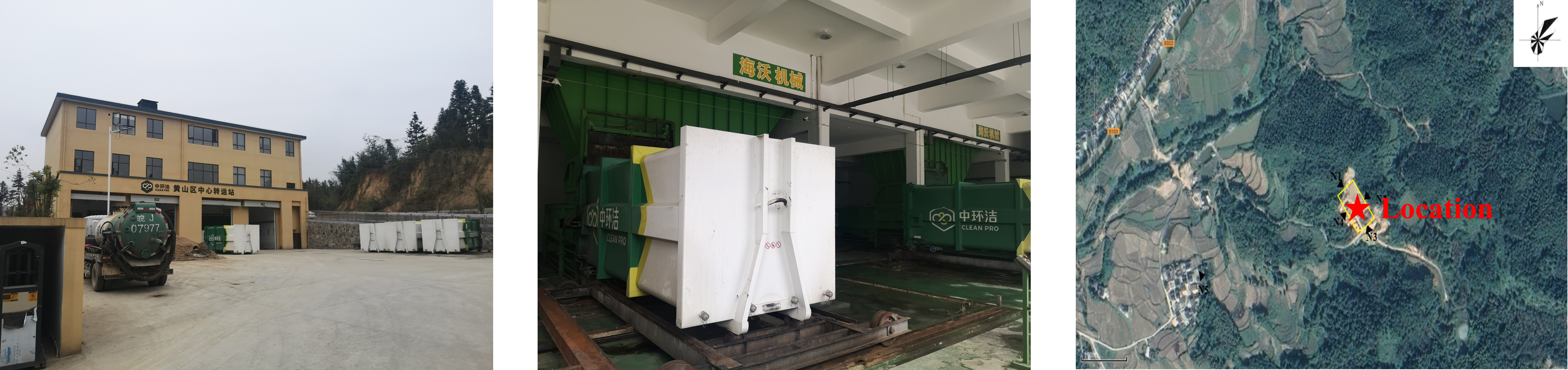


Fig. S1. Scene photos and geographic location of the refuse transfer station


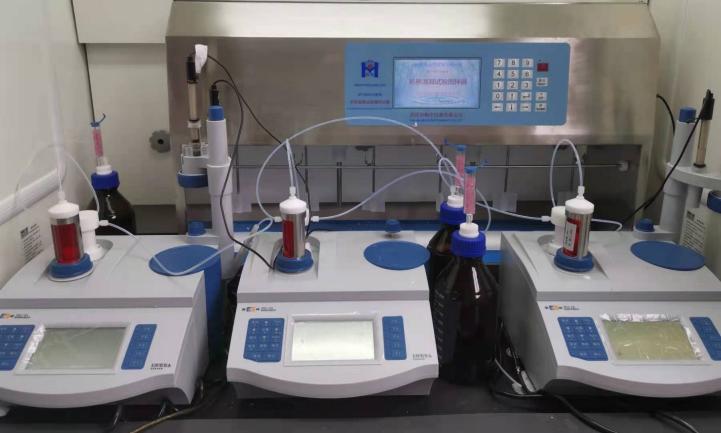


Fig. S2. Photo of batch experimental device





Fig. S3. Adsorption-desorption isotherm of (a) MFB, (b) CSB.


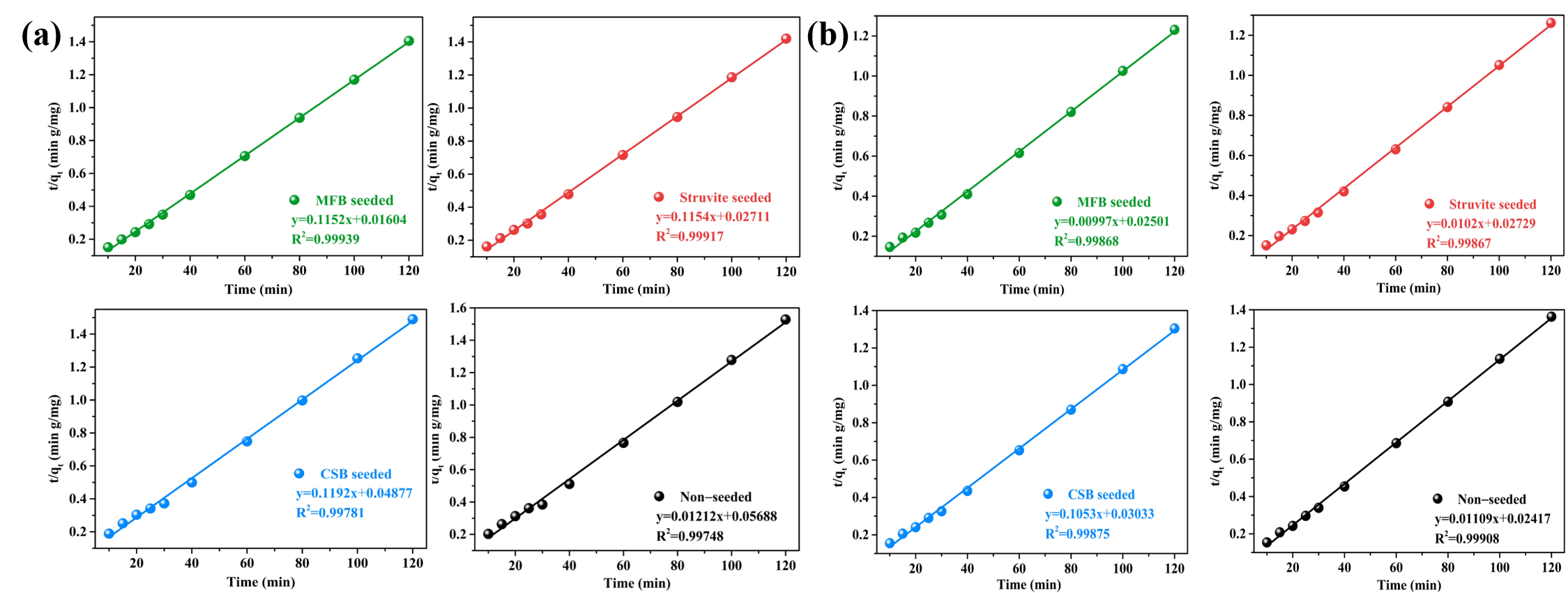


Fig. S4. Pseudo-second-order kinetic fitting of (a) N recovery; (b) P recovery.

**2. Tables**

Table S1. Volatile Organic Compounds (VOCs) content of laeachate

| Parameter | Concentration (μg L^-1^) |
| --- | --- |
| 1,2-Dichloroethane | 348 |
| Ethanol | 0.0000215 |
| Methyl tert-butyl ether | 334 |
| Ethyl acetate | 0.00843 |

Tested by a single quadrupole gas chromatography-mass spectrometer (GCMS-QP2020NX; Shimadzu Scientific Instruments Inc., Japan).

The following VOCs were below the detection limit: Benzene, Toluene, O-xylene, P/m-xylene, 2-chlorotoluene, 4-chlorotoluene, P-Cymene, 1,3,5-Trimethylbenzene, 1,2,4-Trimethylbenzene, Ethylbenzene, Styrene, Cumene, Bromobenzene, Bromoform, Dichloromethane, Chloroform, N-propylbenzene, N-butylbenzene, Sec-butylbenzene, Tert-butylbenzene, Carbon tetrachloride, Bromochloromethane, Bromodichloromethane, Chlorodibromomethane, Dibromomethane, 1,1-Dichloroethane, 1,1,1-Trichloroethane, 1,1,2-Trichloroethane, 1,2-Dibromoethane, 1,1,1,2-Tetrachloroethane, 1,1,2,2-Tetrachloroethane, 1,1,2-Trichloropropane, 1,2-Dibromo-3-chloropropane, 1,2-Dichloropropane, 1,3-Dichloropropane, 2,2-Dichloropropane, 1,2,3-Trichloropropane, chlorobenzene, 1,2-Dichlorobenzene, 1,3-Dichlorobenzene, 1,4-Dichlorobenzene, 1,2,3-Trichlorobenzene, 1,2,4-Trichlorobenzene, 1,1-Dichloroethylene, Cis-1,2-dichloroethylene, Trans-1,2-Dichloroethylene, Trichloroethylene, Perchloroethylene, 1,1-dichloropropene, Cis-1,3-dichloropropene, Trans-1,3-dichloropropene, Hexachloro-1,3-butadiene, Naphthalene, Tert-Butanol, Diisopropyl ether, Ethyl tert-butyl ether, Methyl tert-amyl ether, Cis-1,4-dichloro-2-butene, Trans-1,4-dichloro-2-butene, 2-chloroethyl vinyl ether, Vinyl acetate, Methyl methacrylate, Dibromochloromethane, Dichlorodifluoromethane, Trichlorofluoromethane, Chloroethane, Pentachloroethane, Vinyl chloride, acetone, 2-butanone, 2-hexanone, 4-methyl-2-pentanone, Dibromoethylene, Carbon disulfide, Pyridine, Acrylonitrile, Acrolein, Tetrahydrofuran, Epichlorohydrin.

Table S2 Linear regression models for nutrient recovery efficiency of MFB-seeded process

| Model | | Predictors | R | R^2^ | R^2^ (adj.) | SE Coef | DW |
| --- | --- | --- | --- | --- | --- | --- | --- |
| NH_4_^+^−N recovery efficiency | Seeding dose | | 0.889 | 0.789 | 0.737 | 1.1759 | 1.451 |
|  | pH | | 0.754 | 0.569 | 0.426 | 2.28699 | 1.645 |
|  | Initial NH_4_^+^−N concentration | | 0.959 | 0.919 | 0.892 | 1.57607 | 1.978 |
| PO_4_^3−^−P recovery efficiency | Seeding dose | | 0.788 | 0.620 | 0.525 | 2.6518 | 1.436 |
|  | pH | | 0.876 | 0.767 | 0.689 | 1.76040 | 1.383 |
|  | Initial NH_4_^+^−N concentration | | 0.966 | 0.933 | 0.911 | 1.2917 | 1.490 |

Table S3 Analysis of variance (ANOVA) for the linear regression model

| Model | Predictors | SS | DF | MS | F | *p* |
| --- | --- | --- | --- | --- | --- | --- |
| NH_4_^+^−N recovery efficiency | Seeding dose |  |  |  |  |  |
|  | Regression | 20.737 | 1 | 20.737 | 14.997 | 0.018 |
|  | Residual error | 5.531 | 4 | 1.383 |  |  |
|  | pH |  |  |  |  |  |
|  | Regression | 20.736 | 1 | 20.736 | 3.965 | 0.141 |
|  | Residual error | 15.691 | 3 | 5.230 |  |  |
|  | Initial NH_4_^+^−N concentration |  |  |  |  |  |
|  | Regression | 84.365 | 1 | 84.365 | 33.963 | 0.010 |
|  | Residual error | 7.452 | 3 | 2.484 |  |  |
| PO_4_^3−^−P recovery efficiency | Seeding dose |  |  |  |  |  |
|  | Regression | 45.927 | 1 | 45.927 | 6.531 | 0.063 |
|  | Residual error | 28.128 | 4 | 7.032 |  |  |
|  | pH |  |  |  |  |  |
|  | Regression | 30.625 | 1 | 30.625 | 9.882 | 0.052 |
|  | Residual error | 9.297 | 3 | 3.099 |  |  |
|  | Initial NH_4_^+^−N concentration |  |  |  |  |  |
|  | Regression | 69.794 | 1 | 69.794 | 41.830 | 0.008 |
|  | Residual error | 5.006 | 3 | 1.669 |  |  |

Table S4 Comparison of nutrient recovery efficiency with other reported treatment for leachate

| Treatment | Processing object | NH_4_^+^−N recovery efficiency | PO_4_^3−^−P recovery efficiency | Reference |
| --- | --- | --- | --- | --- |
| Struvite precipitation (Non-seeded) | Rural RTS leachate | 78.5% | 88% | This study |
| Struvite precipitation (MFB seeded) | Rual RTS leachate | 85.4% | 97.5% | This study |
| Struvite precipitation (Magnesite as Mg souce) | Landfill leachate | 28% | 64% | (Warmadewanthi et al., 2021) |
| Struvite precipitation (Stabilizing as precursor) | Landfill leachate | 83% | / | (Huang et al., 2016) |
| Membrane photobioreactor | Landfill leachate | 50% | 70% | (Chang et al., 2018) |
| Biological nitrogen process | Landfill leachate | 84% | / | (Sun et al., 2020) |
| Microbial fuel cell | Landfill leachate | 76.4% | 86.3% | (Nguyen and Min, 2020) |

Table S5. Heavy metals limit in fertilizers among various countries

| Metal | Unit | US | EU | CHN | JPN |
| --- | --- | --- | --- | --- | --- |
| Zn | mg kg^-1^ | 420 | 800 | - | - |
| Cu | mg kg^-1^ | - | 300 | - | - |
| Pb | mg kg^-1^ | 61 | 120 | 200 | 100 |
| Cd | mg kg^-1^ | 10 | 1.5 | 10 | 5 |
| Cr | mg kg^-1^ | - | 2 | 500 | 500 |
| Ni | mg kg^-1^ | 250 | 50 | - | 300 |
| Hg | mg kg^-1^ | 1 | 1 | 5 | 2 |

US: Association of American Plant Food Control Officials (Statement of Uniform Interpretation and Policy (SUIP) #25 - The “Heavy Metal Rule”); EU: the Fertilising Products Regulation 2019/1009 (Regulation (EU) 2019/1009)； CHN: Standardization Administration of China (GB/T 23349-2020); JPN: Ministry of Agriculture, Forestry and Fisheries of Japan (2015).

Table S6 Economic analysis of MFB seeded struvite precipitation process

| Parameter | Unit price | Quantity | Total price |
| --- | --- | --- | --- |
| Mealworm frass | $0.6/kg | 1 kg | $0.6 |
| Magnesium chloride | $0.08/kg | 6.24 kg | $0.5 |
| Sodium dihydrogen phosphate | $0.4/kg | 3.33 kg | $1.33 |
| Sodium hydroxide | $0.25/kg | 3 kg | $0.75 |
| Water | $0.4/ton | 0.1 ton | $0.04 |
| Electricity | $0.1/kW·h | 20 kW·h | $2 |
| Struvite product | 0.657/kg | 7.5 | $4.73 |

Reference

CHANG, H., QUAN, X., ZHONG, N., ZHANG, Z., LU, C., LI, G., CHENG, Z. & YANG, L. 2018. High-efficiency nutrients reclamation from landfill leachate by microalgae Chlorella vulgaris in membrane photobioreactor for bio-lipid production. *Bioresource Technology,* 266**,** 374-381.

HUANG, H., LIU, J., XU, C. & GAO, F. 2016. Recycling struvite pyrolysate obtained at negative pressure for ammonia nitrogen removal from landfill leachate. *Chemical Engineering Journal,* 284**,** 1204-1211.

NGUYEN, H. T. H. & MIN, B. 2020. Leachate treatment and electricity generation using an algae-cathode microbial fuel cell with continuous flow through the chambers in series. *Science of The Total Environment,* 723**,** 138054.

SUN, F., ZHANG, W., JIANG, G., LIU, Y., WU, S., WU, D., SU, X., CHEN, J., LIN, H. & ZHOU, Y. 2020. Effective biological nitrogen process and nitrous oxide emission characteristics for the treatment of landfill leachate with low carbon-to-nitrogen ratio. *Journal of Cleaner Production,* 268**,** 122289.

WARMADEWANTHI, I. D. A. A., ZULKARNAIN, M. A., IKHLAS, N., KURNIAWAN, S. B. & ABDULLAH, S. R. S. 2021. Struvite precipitation as pretreatment method of mature landfill leachate. *Bioresource Technology Reports,* 15**,** 100792.
